# Interfacial Water Coordination on Ruthenium Oxide Nanoparticles Confined Within Covalent Organic Framework and Its Effect on Electrochemical Nitrate Reduction

**DOI:** 10.1002/advs.76147

**Published:** 2026-06-17

**Authors:** Yun Li, Xin Zhao, Arsenii S. Portniagin, Yuxuan Wu, Desui Chen, Haochen Liu, Andrey L. Rogach

**Affiliations:** ^1^ Department of Materials Science and Engineering City University of Hong Kong Kowloon Hong Kong SAR P. R. China; ^2^ Department of Materials Science and Engineering Hainan University Haikou P. R. China; ^3^ Department of Chemistry City University of Hong Kong Kowloon Hong Kong SAR P. R. China

**Keywords:** ammonia production, confined nanoparticle, covalent organic framework, ruthenium oxide, water coordination

## Abstract

Electrochemical nitrate reduction reaction (NO_3_RR) enables sustainable and decentralized ammonia production. Here, we demonstrate how oxygen‐deficient ruthenium oxide (RuO_x_) nanoparticles confined within the imine‐based covalent organic framework (COF) produced from 1,3,5‐tris(4‐aminophenyl)benzene (TAPB) achieve highly efficient nitrate‐to‐ammonia conversion, delivering a high Faradaic efficiency of 99.2% at ‐1.1 V vs Ag/AgCl in a neutral electrolyte. Crystal structure, optical spectra, and electronic state analysis reveal the strong interaction between RuO_x_ nanoparticles and TAPB‐COF. The confined nanoparticles change the interlayer spacing of TAPB‐COF, which in turn results in the high oxygen‐deficiency of RuO_x_. Investigations by in situ optical spectroscopies and ab initio molecular dynamics simulations reveal the occurrence of a repelling effect on water molecules at the surface of hydrophobic TAPB‐COF framework, which contributes to the prevalence of 2‐coordinated water at the surface of RuO_x_ nanoparticles. This ensures a slow‐down proton transfer kinetics, leading to suppression of the hydrogen generation as an undesired competing process. The upshift of d‐band center and bridge‐site adsorption due to the high oxygen‐deficiency and the shortened Ru−Ru distance of the confined RuO_x_ nanoparticles contribute to the strengthened bonding of *NO_2_ and *NOH intermediates, which alleviates the nitrite production and accelerates the NO_3_RR process.

## Graphical abstract

We demonstrate how oxygen‐deficient ruthenium oxide (RuO_x_) nanoparticles confined within covalent organic framework achieve highly efficient nitrate‐to‐ammonia conversion. Strong interaction between RuO_x_ nanoparticles and COF results in modified interfacial hydrogen‐bond water network and adsorption of nitrogenous intermediates. The optimal electrocatalyst delivers an ammonia Faradaic efficiency of 99.2% and exhibits stable operation for over 100 h in a neutral electrolyte.

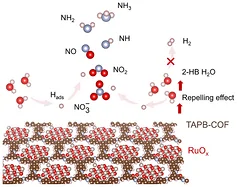

## 1 Introduction

Ammonia is a versatile compound, widely used in chemical synthesis, fertilizers, fuels and clean energy carriers [1]. Electrochemical nitrate reduction reaction, NO_3_^−^+8e^−^+9H^+^→NH_3_+3H_2_O (hereafter abbreviated as NO_3_RR), has emerged as a clean energy route for decentralized ammonia production. The use of excessive nitrate from overfertilization and industrial sewage as a raw source for NO_3_RR not only makes it cost‐saving but also promotes global nitrogen circulation [2]. In comparison with the Harber‐Bosch synthesis, where steam‐reformed hydrogen reacts with nitrogen under elevated temperature (≈500°C) and pressure (> 100 atm), NO_3_RR is more thermodynamically favored owing to the lower N═O bond energy (204 kJ mol^−1^) [2]. However, NO_3_RR experiences a sluggish kinetics due to its complex tandem reaction, involving eight‐electron and nine‐proton transfer processes [3].

Development of electrocatalysts for NO_3_RR relies on the mechanism study to resolve their interactions with nitrogenous intermediates and interfacial water network in the electric double layer [4]. The valence state [5] and the nature of adsorption sites (atop, bridge, and hollow sites) [6] depend on the coordination environment of electrocatalyst and determine the intermediate adsorption. Interfacial microenvironment of electrocatalyst has a significant influence on the proton transfer kinetics of NO_3_RR: while the acceleration of the hydrogen transfer facilitates the protonation process of nitrogenous intermediates, it also speeds up the competitive (and undesirable) hydrogen evolution reaction (HER) [7]. In aqueous environment, hydrogen‐bond H_2_O networks govern the hydrogen transfer rate. Recent studies revealed how different cations (such as Li^+^, Na^+^, and K^+^) and incorporation of organic solvent affect the coordination state of interfacial water and influence the electrocatalytic performance of different reactions, including water splitting [8], carbon dioxide reduction [9], and NO_3_RR process [10]. However, the ability of surface topology of electrocatalysts to tune the interaction with interfacial water molecules has rarely been studied [11].

Herein, we demonstrate how oxygen‐deficient ruthenium oxide (RuO_x_) nanoparticles confined within covalent organic framework (COF) produced from 1,3,5‐tris(4‐aminophenyl)benzene (TAPB) can achieve highly efficient nitrate‐to‐ammonia conversion. Strong interaction between RuO_x_ nanoparticles and TAPB‐COF results in modified interfacial hydrogen‐bond water network and adsorption of nitrogenous intermediates. Through in situ Fourier transform infrared (FTIR) and in situ Raman spectroscopy, as well ab initio molecular dynamics simulations, we established the mechanistic relation between hydrophilicity of the components of the obtained composite and ammonia electrosynthesis efficiency based on interfacial water coordination within the hydrogen‐bond network. Specifically, we found that the repelling effect of hydrophobic TAPB‐COF framework on water molecules contributes to the prevalence of 2‐coordinated water at the surface of RuO_x_ nanoparticles. This effect slows down the proton transfer kinetics during reduction process and thus inhibits the competitive HER process. Dual‐site adsorption supported by the shortened Ru−Ru distance and the upshift of the d‐band center due to the high oxygen‐deficiency of RuO_x_ nanoparticles lead to strong bonding of *NO_2_ and *NOH intermediates, which alleviates the nitrite desorption and accelerates the NO_3_RR process. The optimal electrocatalyst delivers an ammonia Faradaic efficiency of 99.2% of at ‐1.1 V vs Ag/AgCl and exhibits a stable operation for over 100 h at ‐1.5 V vs Ag/AgCl in a neutral electrolyte. Our study demonstrates the feasibility of adjusting the interfacial water microenvironment mediated by the surface topology of electrocatalysts to manipulate their electrochemical behavior.

## 2 Results and Discussion

The formation of oxygen‐deficient RuO_x_ nanoparticles was realized by using the confinement effect of periodic pore structure in TAPB‐COF, where the imine‐based organic framework was chosen as the template because of the reported high crystallinity [12, 13, 14]. TAPB and 2‐hydroxy‐1,3,5‐triformylbenzene (HTFB) were used as monomers to synthesize TAPB‐COF through Schiff‐base reaction, where the resulting imine nitrogen and hydroxyl group (salicylaldehyde‐imine unit) of HTFB form 6‐membered chelates with metal cations and uniformly disperse them over the TAPB‐COF framework (Figure S1). To ensure well‐developed crystal structure of COF, 1,2‐diaminocyclohexane and deionized water were introduced to promote the reversibility of this reaction. TAPB‐COF acted as a nanoreactor for nucleation of RuO_x_ nanoparticles, where the imine and hydroxyl groups chelated Ru^3+^ cations from RuCl_3_ precursor. The formation of RuO_x_ nanoparticles proceeded without adding any buffer, as the strong Lewis acid property of Ru^3+^ accelerated the deprotonation of coordinated water molecules; while the periodical pore structure of TAPB‐COF restrained their growth and induced high‐dense atomic defects. In the following discussion, we will denote the obtained composite materials (RuO_x_ nanoparticles confined within TAPB‐COF, where the Ru amount is determined to be 7.46 wt%) as RuO_x_@TAPB‐COF; we will compare them with two reference samples, TAPB‐COF itself and commercial RuO_2_ nanoparticles.

**FIGURE 1:**
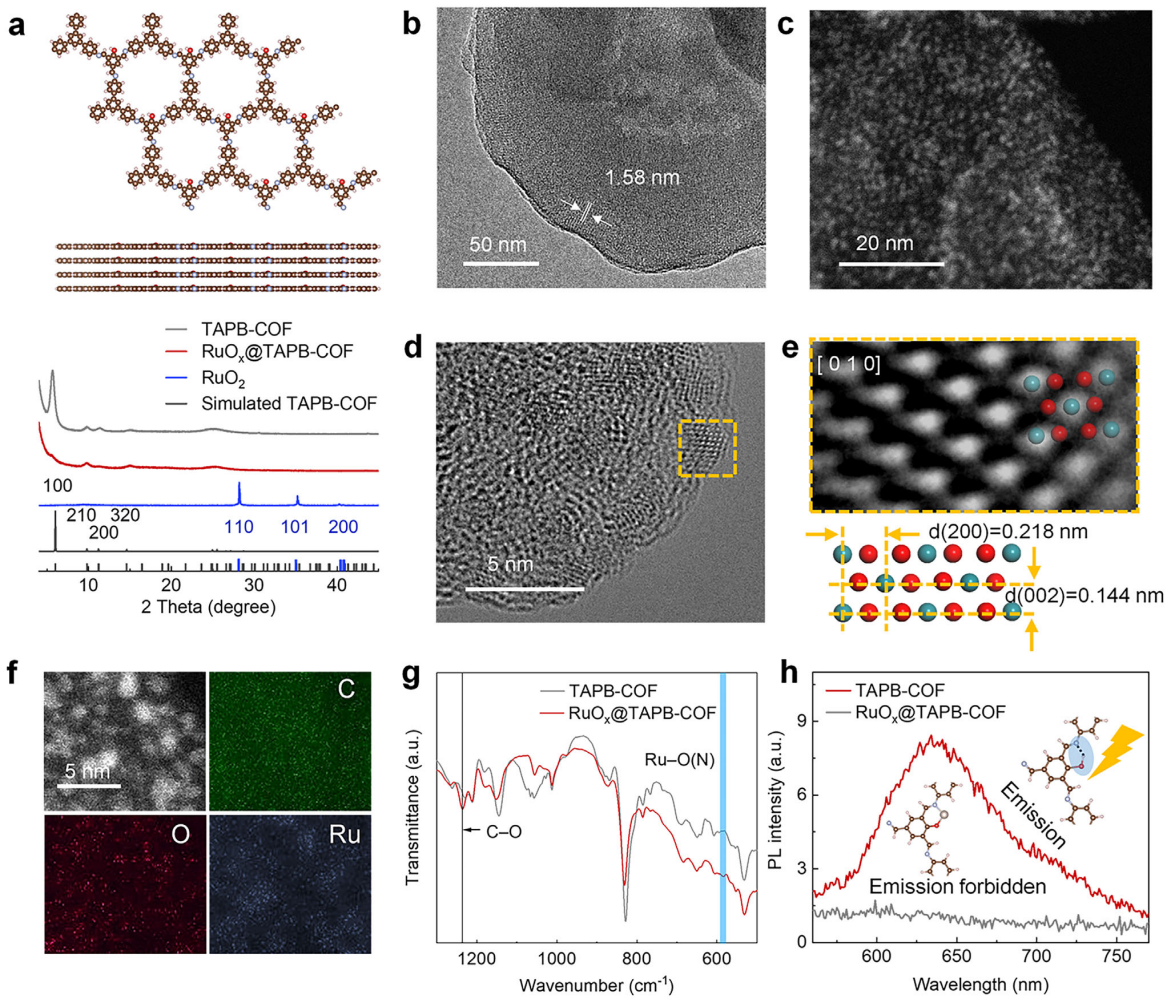
Characterization of TAPB‐COF, RuO_x_@TAPB, and RuO_2_. (a) Molecular structure of TAPB‐COF, and powder XRD patterns of the three samples. The dark grey line pattern at the bottom represents simulated Bragg diffraction peaks for TAPB‐COF, while the blue line pattern belongs to rutile RuO_2_ (PDF# 00‐018‐1139). (b) HRTEM image of TAPB‐COF produced by Schiff‐base reaction. (c) HAADF‐STEM image, (d) HRTEM image, and (e) inverse Fourier transformed image demonstrating formation of RuO_x_ nanoparticles within TAPB‐COF. The inverse Fourier transformed image in (e) was obtained from the area within yellow square of spherical aberration‐corrected HRTEM image in (d); the crystal structure of rutile RuO_2_ projected along the [0 1 0] zone axis, where cyan and red spheres represent Ru and O, respectively, is shown underneath. (f) Elemental mapping for C, O, and Ru in a fragment of RuO_x_@TAPB‐COF. (g) FTIR and (h) PL spectra of TAPB‐COF and RuO_x_@TAPB‐COF. The salicylaldehyde‐imine unit (dark blue region in the inset of Figure 1h) contributes to the red emission, while the chelation of Ru cation replacing the H atom results in the absence of emission. Color coding for the elements: Ru, brown; O, red; H, white; C, dark brown; N, light blue.

Powder X‐ray diffraction (XRD) pattern (Figure 1a) indicates high crystallinity of TAPB‐COF with diffraction peaks at 2*θ* = 5.7 °, 9.8 °, 11.4 °, 15.2 °, and 25.2 °belonging to (1 0 0), (2 1 0), (2 0 0), (3 2 0), and (0 0 1) planes, respectively. This is consistent with the simulated XRD results (Figure 1a), for which details of the simulated lattice structure of TAPB‐COF are provided in Figure S2. The diffraction peaks for (1 0 0), (2 0 0), (2 1 0), (3 1 0), and (0 0 1) planes were also observed in RuO_x_@TAPB‐COF, where the intense (2 1 0) diffraction indicated the well‐maintained TAPB‐COF framework after formation of RuO_x_ nanoparticles. However, the diffraction peaks for (1 0 0) and (2 0 0) plane became weakened, which could be attributed to the out‐of‐plane twist of the salicylaldehyde‐imine unit driven by the strong chelation of Ru cations within the pore‐confined RuO_x_ nanoparticles. The missing diffraction peaks for RuO_2_ phase in the RuO_x_@TAPB‐COF composite can be due to the low crystallinity and ultrasmall size of RuO_x_ nanoparticles.

Scanning electron microscopy (SEM) and high angle annular dark field scanning transmission electron microscopy (HAADF‐STEM) images (Figures S3 and S4, respectively) show hollow TAPB‐COF disks with a diameter around 150 nm. High resolution transmission electron microscopy (HRTEM) image (Figure 1b) demonstrates the lattice spacing of 1.58 nm assigned to the (1 0 0) plane of TAPB‐COF, consistent with the XRD data. The disappearance of stretching bands for C═O at 1685 cm^−1^ from aldehyde group in HTFB and C−N at 1280 cm^−1^ from amine group in TAPB, and appearance of a new characteristic vibration of C═N at 1216 cm^−1^ in FTIR spectrum (Figure S5) indicates that the resulting TAPB‐COF framework is linked through the new‐formed imine bond as a result of the Schiff‐base reaction. Multiple RuO_x_ nanoparticles with a size of 2.0 ± 0.2 nm (Figure S6) can be well‐recognized within the RuO_x_@TAPB‐COF composite in HRTEM and HAADF‐STEM images, as illustrated in Figures S7 and S8, and Figure 1c. HRTEM image (Figure 1d) and inverse Fourier transformed image (Figure 1e) demonstrate the lattice fringes with 0.218 and 0.144 nm lattice spacing corresponding to (2 0 0) and (0 0 2) plane of RuO_2_ tetragonal phase (PDF#00‐018‐1139), which further corroborates successful integration of RuO_x_ nanoparticles within the COF framework. Accordingly, (1 1 0) lattice plane was observed in the reference sample (pristine RuO_2_ nanoparticles with the size of ≈10 nm) as shown in Figure S10a, where the lattice spacing is 0.314 nm (Figure S10b). Electron energy loss spectra of RuO_x_@TAPB‐COF measured in two different regions (with and without RuO_x_ nanoparticles, as shown in Figure S11a) demonstrate the typical C‐K edge belonging to the TAPB‐COF at both regions, while the characteristic peak of Ru‐M_2,3_ edge is only observed at the region with nanoparticle (site 1) in RuO_x_@TAPB‐COF (Figure S11b) [15, 16, 17]. Energy dispersive spectroscopy (EDS) provided elemental mapping for C, O, and Ru in RuO_x_@TAPB‐COF (Figure 1f), demonstrating an even distribution of RuO_x_ nanoparticles within the TAPB‐COF framework.

To verify the chelation of Ru cation within RuO_x_@TAPB‐COF, we have compared FTIR and Raman spectra of pristine RuO_2_, TAPB‐COF, and RuO_x_@TAPB‐COF. As illustrated in Figure 1g, blue‐shift of C−O stretching vibrations (*δ* = 6 cm^−1^) and appearance of a new vibration of Ru−O(N) at 585 cm^−1^ in the FTIR spectrum of RuO_x_@TAPB‐COF as compared to TAPB‐COF points on the coordination of Ru cation through imine and hydroxyl groups [18]. Raman spectra in Figure S12 revealed three characteristic peaks at 1172.8, 1371.2 and 1580.5 cm^−1^, which can be assigned to the vibrational peaks of C−H, −C═C− and −C═N− of TAPB‐COF [19, 20]. Additionally, the characteristic vibration of Ru−O(N) was observed around 560 cm^−1^ as a broad peak, indicating some lattice imperfections within RuO_x_ nanoparticles confined in TAPB‐COF [21]. Salicylaldehyde‐imine unit (blue region marked in the inset of Figure 1h) can undergo proton migration from O to N via the fixed hydrogen bond under excitation, forming a keto‑enol tautomer that decays radiatively, giving rise to a Stokes‑shifted fluorescence [22]. Comparing the PL spectra of TAPB‐COF and RuO_x_@TAPB‐COF (Figure 1h), one can see that TAPB‐COF shows an emission centered at 634 nm, whereas this peak is missing in RuO_x_@TAPB‐COF, certifying on the coordination of Ru with O/N (Ru−O(N)), since the proton transfer involved excitation is forbidden.

**FIGURE 2:**
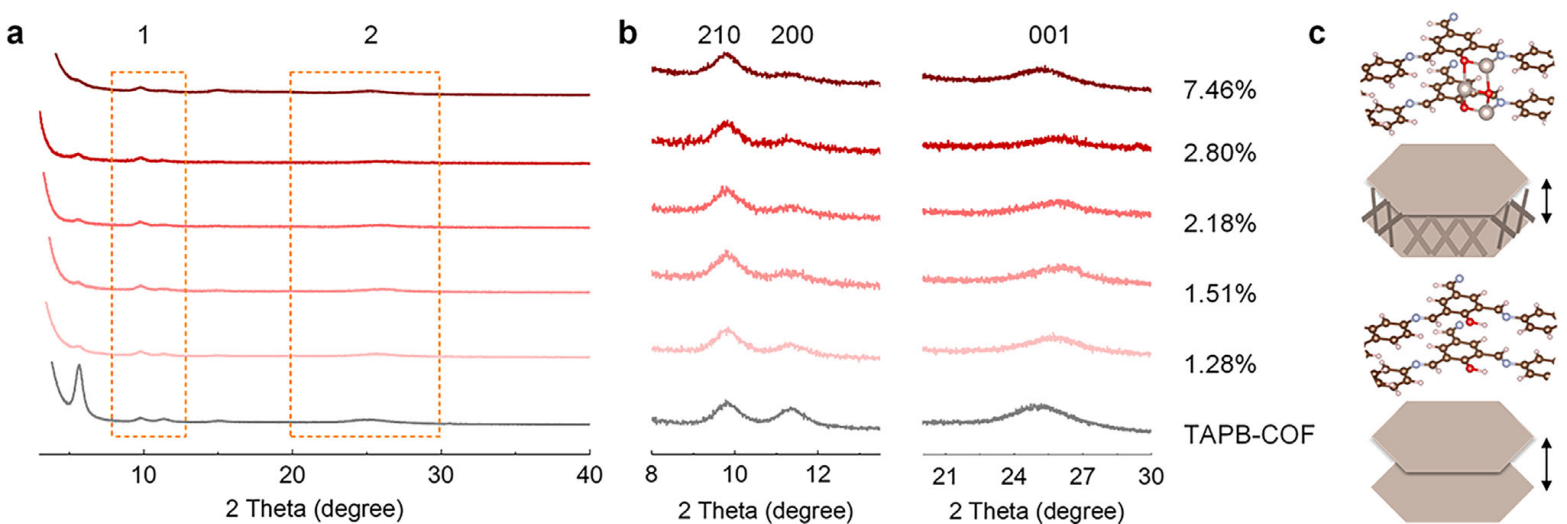
Structural evolution of TAPB‐COF upon formation of RuO_x_ nanoparticles. (a) Powder XRD patterns of RuO_x_@TAPB‐COF samples with varying Ru amount (wt%) of 1.28%, 1.51%, 2.18%, 2.80%, and 7.46%, and (b) enlarged XRD patterns of regions within the two orange squares 1 and 2 from the frame (a). (c) Schematic diagram illustrates the effect of RuO_x_ nanoparticles acting as a “tailor” on the interlayer spacing of TAPB‐COF.

The weakened Bragg diffraction of (1 0 0) and (2 0 0) lattice planes of TAPB‐COF after formation of RuO_x_ nanoparticles can be attributed to the dragging force of the latter, which is strong enough to trigger the structural deformation of the TAPB‐COF framework. We synthesized a series of RuO_x_@TAPB‐COF samples with varying Ru amount (wt%) of 1.28%, 1.51%, 2.18%, 2.80%, and 7.46%, and analyzed its effect on the lattice structure of TAPB‐COF. As seen in Figure S13, SEM images show that the hollow disk shape of TAPB‐COF was well‐maintained in all these five samples, and there were no larger‐sized RuO_x_ nanoparticles at the surface. XRD patterns in Figure 2a reveal the coexistence of (1 0 0), (2 1 0), (2 0 0), and (0 0 1) planes in these five samples. Consistently, the peak intensity of the (2 1 0) plane showed almost no change, while the intensity of the (2 0 0) plane gradually decreased upon increasing of the Ru amount from 1.28 to 7.46 wt%. Moreover, a peak shift occurred for the (0 0 1) plane (Figure 2b), which is related to the changing interlayer distance of TAPB‐COF as depicted in Figure S2. According to the Bragg's law, we calculated the lattice spacing of (0 0 1) planes of RuO_x_@TAPB‐COF with different Ru amount. As shown in Figure S14, with an increase of loading to 1.51 wt%, the interlayer distance decreased from 3.56 to 3.43 Å and then returned to 3.55 Å at a loading of 7.46 wt%. PL spectra in Figure S15 reveal that the red emission was forbidden in all five RuO_x_@TAPB‐COF samples, reflecting chelation of Ru with the salicylaldehyde‐imine unit. We also measured the nitrogen adsorption‐desorption isotherms for TAPB‐COF and RuO_x_@TAPB‐COF with Ru loading of 7.46 wt%. The isotherms revealed coexistence of micropores and mesopores in TAPB‐COF indicative by the H4 type hysteresis curves (Figure S16a). The BET surface areas of the TAPB‐COF and RuO_x_@TAPB‐COF were determined to be 695.5 and 388.5 m^2^ g^−1^, indicating the decrease of specific surface area of TAPB‐COF after formation of RuO_x_ nanoparticles. The pore size distribution of pristine TAPB‐COF (Figure S16b) ranged from 0.85 to 2.20 nm, and there was also a fraction of pores ranging from 2.20 to 3.70 nm. The existence of larger pores can be attributed to the imperfect crystallization of TAPB‐COF. The size distribution of RuO_x_ nanoparticles in RuO_x_@TAPB‐COF is centered at 2.0 ± 0.2 nm (Figure S6), which coincides with the pore size distribution range of TAPB‐COF. The weakened intensity of the two peaks in the RuO_x_@TAPB‐COF sample (Figure S16b) reflects the occupying of RuO_x_ nanoparticles within those pores of TAPB‐COF. Based on these observations, the coordination of Ru cations may occur first and link the adjacent COF layers; then the RuO_x_ nanoparticles confined within the TAPB‐COF are formed, acting as a “tailor” to sew up the adjacent TAPB‐COF layers, as illustrated in Figure 2c, resulting in the out‐of‐plane twist of each single layer and shrinkage of the interlayer distance. When the RuO_x_ nanoparticles become large enough, they expand the space, leading to the recovery of the interlayer distance. In this case, the diffraction from (1 0 0) and (2 0 0) planes became weakened, while the peak of the (0 0 1) plane initially shifted towards a high angle and then gradually moved back toward its original position (Figure 2b).

**FIGURE 3:**
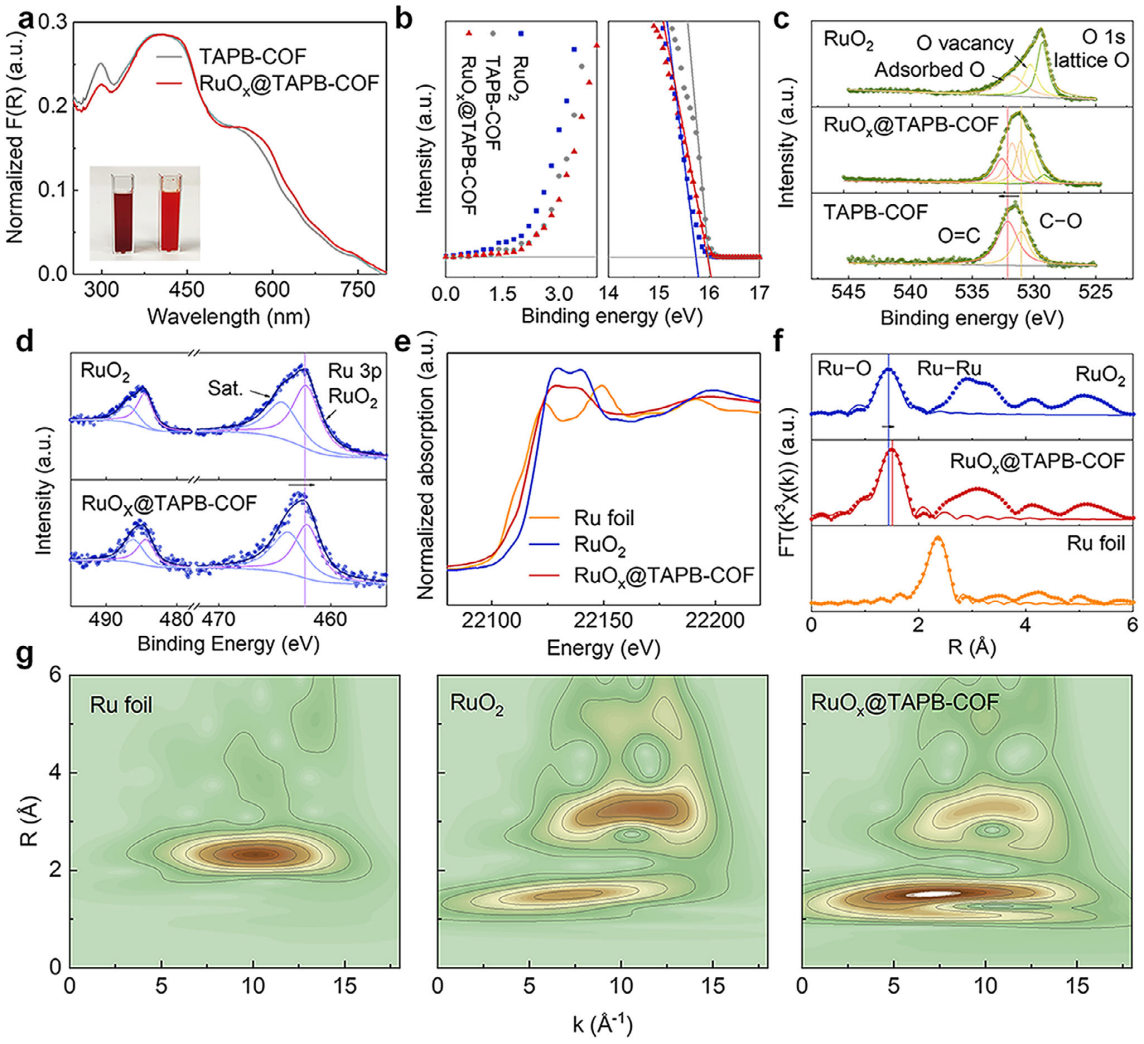
Characteristics of RuO_x_@TAPB‐COF, compared with the reference RuO_2_ and TAPB‐COF samples. (a) UV–vis absorption spectra of TAPB‐COF and RuO_x_@TAPB‐COF; the inset shows photograph of colloidal suspensions of RuO_x_@TAPB‐COF (left) and TAPB‐COF (right). (b) UPS spectra and (c) high‐resolution XPS spectra of O 1s orbitals of RuO_2_, TAPB‐COF, and RuO_x_@TAPB‐COF. (d) XPS spectra of Ru 3p orbitals for RuO_2_ and RuO_x_@TAPB‐COF. (e) XANES spectra, (f) EXAFS spectra and (g) wavelet transform contour plots of the Ru foil, RuO_2_, and RuO_x_@TAPB‐COF.

As shown in the inset of Figure 3a, the color of colloidal suspensions changed from bright red to crimson upon formation of RuO_x_ nanoparticles within TAPB‐COF. A red‐shift of 19 nm occurred for the first exciton absorption peak of RuO_x_@TAPB‐COF comparing with that of the pristine TAPB‐COF. To clarify the electronic interaction between RuO_x_ nanoparticles and TAPB‐COF, ultraviolet photoelectron spectroscopy (UPS) has been performed. The observed higher work function (5.23 eV) in RuO_x_@TAPB‐COF in comparison with *n*‐type TAPB‐COF (5.21 eV) is indicative of the electron transfer occurring from TAPB‐COF to RuO_x_ nanoparticles (Figure 3b). This value is lower than the work function (5.46 eV) determined for RuO_2_ sample, revealing the enhanced electron donating property of RuO_x_ nanoparticles within the RuO_x_@TAPB‐COF. X‐ray photoelectron spectroscopy (XPS) and X‐ray absorption fine structure measurements were further conducted to analyze the electronic states and the local coordination environment of Ru in RuO_x_@TAPB‐COF, whereas pristine TAPB‐COF and RuO_2_ samples served as reference samples for comparison. The deconvoluted O 1s peaks in XPS spectra presented in Figure 3c reveal coexistence of O═C (benzoquinone‐state) and C−O (phenol‐state) in TAPB‐COF [23], which shifted towards higher binding energy in RuO_x_@TAPB‐COF. This indicates the electron loss of O in benzoquinone state and phenol state upon coordination with Ru from RuO_x_ nanoparticles. Importantly, high density of the oxygen defects was found in RuO_x_@TAPB‐COF, where the ratio between the oxygen vacancies and lattice oxygen was determined as 3.53:1 by integrating deconvoluted O 1s peaks; this ratio was much higher than that in pristine RuO_2_ (0.79:1). Accordingly, the Ru 3p XPS peak (Figure 3d) shifted from 462.41 eV in RuO_2_ to 462.18 eV in RuO_x_@TAPB‐COF, implying the lower oxidation state of Ru as a result of the high density of the oxygen vacancies and electron transfer from the TAPB‐COF [24, 25]. This was further confirmed by the negative shift of Ru 3d XPS peaks in the case of RuO_x_@TAPB‐COF as compared to RuO_2_ sample (Figure S17). We further studied the oxidation state of Ru in RuO_2_ and RuO_x_@TAPB‐COF using X‐ray absorption near‐edge structure (XANES) spectroscopy. As shown in Figure 3e, the oxidation state of Ru in RuO_x_@TAPB‐COF was indeed lower than that in RuO_2_, consistent with the XPS data. Extend X‐ray absorption fine structure (EXAFS) spectra shown in Figure 3f certify on the longer Ru−O bond length of 1.99 Å in the RuO_x_@TAPB‐COF, compared to 1.96 Å in the RuO_2_. The extended Ru−O length is attributed to reduced oxidation state of Ru induced by the high density of oxygen defects in RuO_x_@TAPB‐COF [26], which would impair the orbital hybridization between the Ru 3d and O 2p orbitals, resulting in a weakened Ru–O bond. Based on the least‐squares EXAFS fitting (Table S1), the coordination number (CN) of Ru in RuO_x_@TAPB‐COF was estimated to be 3.2, which was significantly less than that of pristine RuO_2_ (5.3). The wavelet transforms contour plots (Figure 3g) show a maximum intensity around 7.35 Å^−1^ in relation to the Ru−O bond in RuO_2_ and RuO_x_@TAPB‐COF. Simultaneously, the Ru−Ru bond was found to be 11.10 Å^−1^ in both samples; its intensity was weaker in RuO_x_@TAPB‐COF than that in RuO_2_ due to the smaller‐sized nanoparticles in the former case. The electron accumulation around RuO_x_ nanoparticles as a result of the charge redistribution within the RuO_x_@TAPB‐COF composite leads to the up‐shift of the d‐band center of Ru sites [27]. As a crucial descriptor, the position of the d‐band center commonly determines the adsorption strength between catalysts and adsorbates. Thus, the Fermi‐level approached d‐band center would strengthen adsorption of critical intermediates, *NH_2_ and *NH_3_, involved in the NO_3_RR process [21]. Moreover, the revealed lower oxidation state of Ru in RuO_x_@TAPB‐COF should enhance the electron donating properties of RuO_x_ nanoparticles, thus facilitating the NO_3_RR process.

**FIGURE 4:**
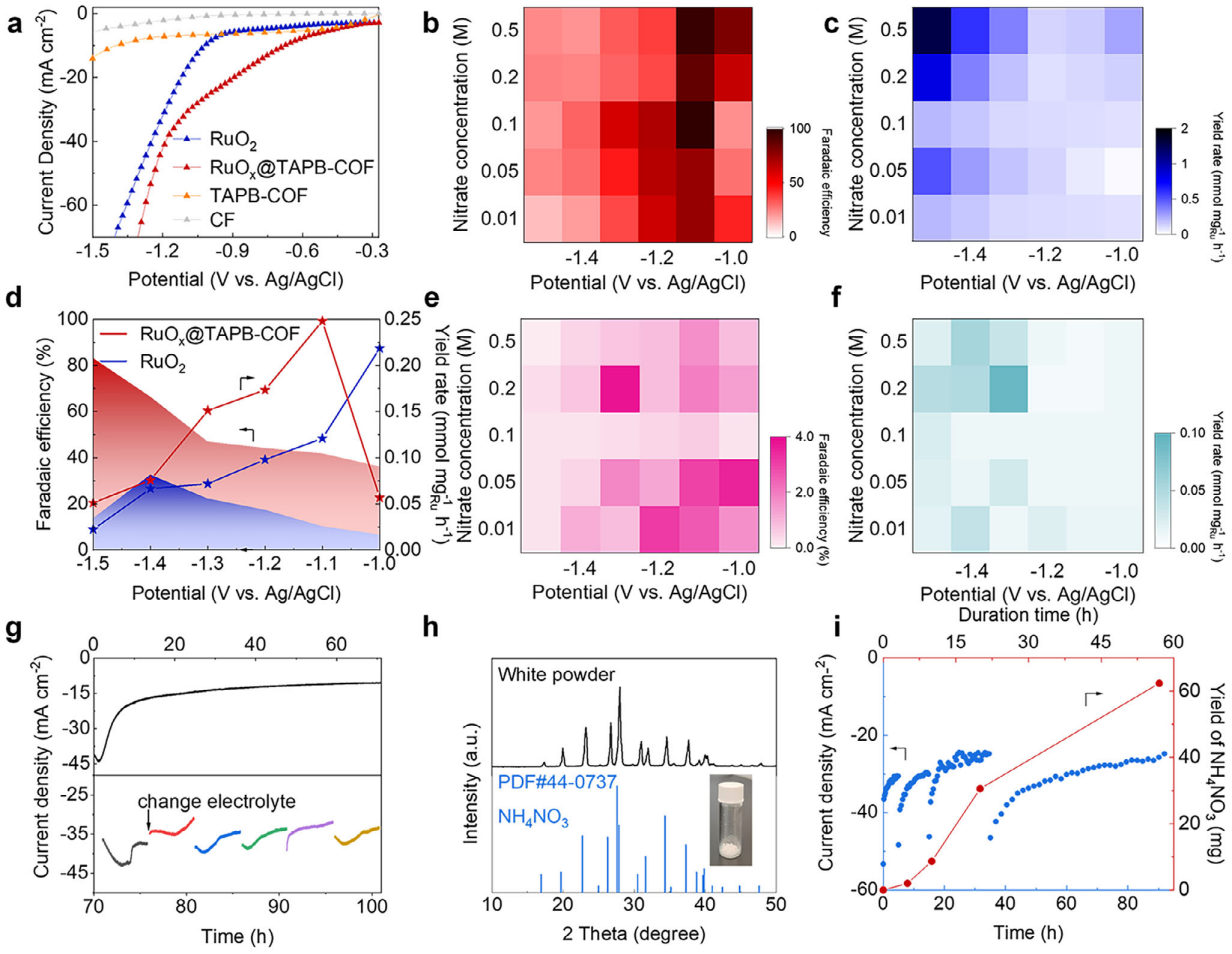
Electrochemical NO_3_RR performance of the RuO_x_@TAPB‐COF electrocatalyst, compared with the reference RuO_2_ and TAPB‐COF samples. (a) LSV curves of RuO_2_, RuO_x_@TAPB‐COF, TAPB‐COF, and carbon felt (CF). (b) Faradaic efficiency and (c) yield rate of ammonium determined for the RuO_x_@TAPB‐COF electrocatalyst under different potentials and nitrate concentrations. (d) Faradaic efficiency and yield rate of ammonium for RuO_2_ and RuO_x_@TAPB‐COF measured in 0.1 m Na_2_SO_4_ with addition of 0.1 m NaNO_3_. (e) Faradaic efficiency and (f) yield rate of nitrite for RuO_x_@TAPB‐COF under different potentials and nitrate concentrations. (g) Duration curves of RuO_x_@TAPB‐COF measured under a constant potential of ‐1.5 V vs Ag/AgCl. In the bottom frame, the electrolyte was changed at each time interval. (h) XRD pattern of the collected NH_4_NO_3_ powder; the line pattern at the bottom corresponds to the PDF#44‐0737 for this substance. (i) Chronopotentiometry curves (in blue) and the yield of NH_4_NO_3_ (in red) for RuO_x_@TAPB‐COF at ‐1.5 V vs Ag/AgCl.

The NO_3_RR performance of the RuO_x_@TAPB‐COF electrocatalyst was investigated in neutral electrolyte with 0.1 m Na_2_SO_4_ and 0.1 m NaNO_3_ using a three‐electrode system. Linear sweep voltammetry (LSV) curves shown in Figure 4a indicate that, in comparison with commercial RuO_2_ electrocatalyst (see Supporting Information for details), there was a prominent current density increase in the case of RuO_x_@TAPB‐COF, especially at a low overpotential range. At the same time, the large polarization suggested the poor NO_3_RR performance of TAPB‐COF, highlighting the role of the interaction between RuO_x_ nanoparticles and TAPB‐COF on improving the electrochemical activity. Differing from the measured HER current density in 0.1 m Na_2_SO_4_, significantly enhanced NO_3_RR current density observed in this electrolyte with addition of 0.1 m NaNO_3_ (Figure S19) revealed the preferential occurrence of NO_3_RR when using RuO_x_@TAPB‐COF as electrocatalyst. We also studied how the applied potential and the concentration of NaNO_3_ influenced the Faradaic efficiency and the yield rate of ammonium in case of RuO_x_@TAPB‐COF. The concentrations of the produced ammonium and nitrite were determined using standard calibration curves by colorimetric method, as demonstrated in Figures S20 and S21, respectively [28]. Figure 4b revealed a volcano relationship between the ammonia Faradaic efficiency and elevated potential. The efficiency exceeded 70% when the concentration of ${\mathrm{NO}}_{3}^{-}$ ranged from 0.01 to 0.5 m at ‐1.1 V vs Ag/AgCl, and the maximum value of 99.2% was observed for 0.1 m NaNO_3_ electrolyte. This value is higher than most of the previously reported electrocatalysts in a neutral electrolyte (Figure S22). The yield rate of ammonia (Figure 4c) was found to scale with both the applied potential and the concentration of nitrate, with the largest yield rate of 1.29 mmol ${\mathrm{mg}}_{\mathrm{Ru}}^{-1}$ h^−1^ in 0.5 m NaNO_3_ when the reaction bore a potential of ‐1.5 V vs Ag/AgCl. We also investigated the yield of nitrite as by‐product during the NO_3_RR process and foundd out that its Faradaic efficiency was less than 4% in RuO_x_@TAPB‐COF (Figure 4e). Figure 4e,f shows both strong potential and nitrate concentration dependence for the Faradaic efficiency and the yield of nitrite. Both low applied bias and low nitrate concentration contributed to the desorption of nitrite intermediate (Figure 4e), whereas more nitrite was produced when these two values increased (Figure 4f).

Additionally, we have compared the potential‐dependent NO_3_RR reactivity of RuO_x_@TAPB‐COF with the reference RuO_2_ sample in 0.1 m NaNO_3_. Figure 4d demonstrates the inferior ammonia Faradaic efficiency of RuO_2_ in a wide potential range (< ‐1.1 V vs Ag/AgCl). Severe bubbles release was observed during the electrolysis using RuO_2_ as electrocatalyst, implying severe competitive HER. RuO_x_@TAPB‐COF exhibited higher mass activity within the whole measured potential range, especially at a large polarization potential. The largest yield rate of ammonia (0.21 mmol ${\mathrm{mg}}_{\mathrm{Ru}}^{-1}$ h^−1^) was observed for RuO_x_@TAPB‐COF at ‐1.5 V vs Ag/AgCl, which was around 3 times higher than that of RuO_2_. The low ammonia Faradaic efficiency of pristine TAPB‐COF (less than 16% under the potential ranging from ‐1.0 to ‐1.5 V vs Ag/AgCl) indicated the synergistic effect between TAPB‐COF and RuO_x_ nanoparticles (Figure S25). Simultaneously, we investigated the ammonia Faradaic efficiency of RuO_x_@TAPB‐COF with different Ru amount under a constant potential of ‐1.1 V vs Ag/AgCl. As illustrated in Figure S26, the efficiency showed a positive correlation with Ru loading, increasing from 18.0% to 99.2% as the Ru loading increased from 1.28 to 7.46 wt%. We also used a ^15^N isotope‐labeling method to track the conversion of nitrate‐to‐ammonium. The observed nuclear magnetic peaks of ^15^NH_4_^+^ when feeding ^15^NO_3_^−^ as the nitrogen source (Figure S27) confirmed that the ammonia production occurred due to conversion of nitrate rather than due to the cleavage of imide bonds in TAPB‐COF.

We recorded the current density–operating time curves for the RuO_x_@TAPB‐COF electrocatalyst. The evolution of current density at ‐1.1 V vs Ag/AgCl over time presented in Figure S28, that there was a slight current decay during 100‐h electrocatalysis, reflected the gradual consumption of nitrate ions in the electrolyte. We then selected the constant potential of ‐1.5 V vs Ag/AgCl to investigate the stability of RuO_x_@TAPB‐COF, where the maximum yield rate of ammonia can be reached. As seen in Figure 4g, there was 24.7 mA cm^−2^ drop of the current density after 10 h under a constant potential ‐1.5 V vs Ag/AgCl. The current density recovered after 70 h operation when replacing the fresh electrolyte, which was confirmed by measuring continuous 6 cycles of 5 h electrolysis under the same potential; this indicated an outstanding NO_3_RR activity and stability of the RuO_x_@TAPB‐COF electrocatalyst. During the electrolysis, we introduced an argon flow to carry generated gas and used nitric acid to trap ammonia; the obtained white powder was collected using rotary evaporator. The XRD pattern of this substance was confirmed to belong to crystalline NH_4_NO_3_ (Figure 4h). We also recorded the yield of the generated NH_4_NO_3_ at different operating times (Figure 4i), which exhibited a positive scaling relation with the extended electrolysis time. The maximal yield rate reached 3.056 mg cm^−2^ h^−1^, meaning that 30.65 mg NH_4_NO_3_ powder was produced within 20 h (Figure 4i).

Zn‐NO_3_RR batteries have emerged as a bifunctional system, which not only provides the electricity supply but also can act as ammonia generators. Thus, we assembled neutral Zn‐NO_3_RR batteries using either RuO_x_@TAPB‐COF or RuO_2_ as the cathode, respectively. As shown in Figure S29, the former battery exhibited an open circuit potential of 1.60 V vs Zn^2+^/Zn, which was higher than that of RuO_2_ based one (1.57 V vs Zn^2+^/Zn). Furthermore, RuO_x_@TAPB‐COF showed higher discharge potential of ≈0.5 V vs Zn^2+^/Zn under constant current density of 0.5 mA cm^−2^ (Figure S30). The discharge potential remained nearly unchanged in the case of RuO_x_@TAPB‐COF, whereas severe deterioration was observed for RuO_2_, indicating the enhanced stability of this electrocatalyst because of the confinement effect of TAPB‐COF on RuO_x_ nanoparticles.

**FIGURE 5:**
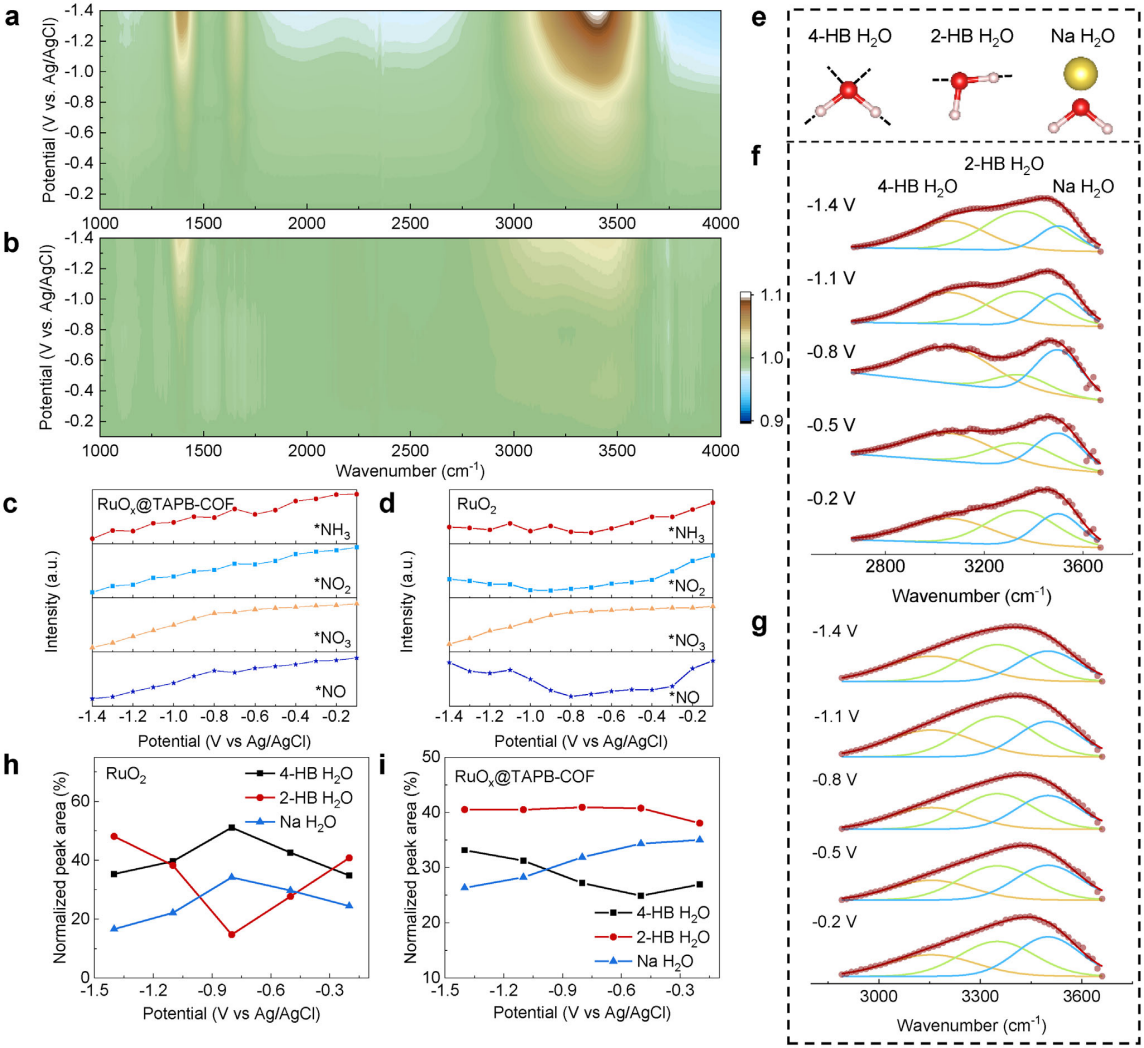
In situ FTIR spectra and the analysis of mechanism of NO_3_RR process. In situ FTIR spectra of (a) RuO_x_@TAPB‐COF and (b) RuO_2_ at different potentials in 0.1 m Na_2_SO_4_ with addition of 0.1 m NaNO_3_. Potential‐dependent intensities of characteristic vibrations of *NH_3_, *NO_2_, *NO_3_, and *NO intermediates during NO_3_RR for (c) RuO_x_@TAPB‐COF and (d) RuO_2_. (e) Atomic models of interfacial water molecules for 4‐HB H_2_O, 2‐HB H_2_O, and Na H_2_O. Color coding for the elements: O, red; H, white, Na, yellow. Deconvoluted FTIR vibrations for O−H stretching mode of interfacial water molecules and variations of relative proportions under elevated potential in (f, h) RuO_2_ and (g, i) RuO_x_@TAPB‐COF.

To elucidate the influence of TAPB‐COF on the confined RuO_x_ nanoparticles in accelerating the NO_3_RR process, in situ FTIR and in situ Raman spectroscopies were employed to monitor the reactants and intermediates. The characteristic peak of the formed NH_3_ at 1125 cm^−1^ gradually increased under elevated working potentials from ‐0.2 to ‐1.4 V vs Ag/AgCl (Figure 5a), indicating the direct conversion of NO_3_^−^ to NH_3_ on RuO_x_@TAPB‐COF [29, 30, 31]. Similarly, the signals of *NO_3_ (1384 cm^−1^), *NO_2_ (1222 cm^−1^) *NO (1664 cm^−1^), and *NH_2_ (1406 cm^−1^) intermediates became gradually enhanced under even higher working potentials [6, 32]. The hydrogenous intermediates (−NH at 1147 cm^−1^, −NH_2_ at 1395 cm^−1^, and NH_3_ at 1526 cm^−1^) were further detected through *in‐situ* Raman measurements (Figure S31a), which also showed potential‐dependent characteristics [33]. When using RuO_2_ as an electrocatalyst, similar FTIR vibration modes and Raman scattering signals reflecting homologous nitrogenous intermediates generated during NO_3_RR were in situ detected, as well (Figure 5b and Figure S31b, respectively). However, there was a different tendency on how the characteristic FTIR peak intensity changed with elevated potential in the case of RuO_x_@TAPB‐COF versus RuO_2_ (Figure 5c,d), which pointed on the enhanced hydrogenation of RuO_x_@TAPB‐COF during the NO_3_RR process. This was evidenced by the appearance of a limited potential at app. ‐0.8 V vs Ag/AgCl in the case of RuO_2_, under which the FTIR peak intensities of *NO, *NO_2_, and *NH_3_ intermediates reached their extreme values, meaning that further increase of polarization was not able to accelerate the NO_3_RR process in spite of the enhanced adsorption of *NO_3_. In contrast, in the case of RuO_x_@TAPB‐COF, these peaks showed a positive correlation with the elevated potential in a wide range from ‐0.2 to ‐1.4 V vs Ag/AgCl, which means the hydrogenation process of NO_3_RR dominated the electrocatalysis rather than the competitive Heyrovsky and Tafel step of HER process.

In neutral electrolytes, hydrogenation process of NO_3_RR mainly relies on the interfacial water network [34]. This network, as detected by FTIR spectroscopy, is formed by hydrogen‐bonded water and alkali metal‐ionized water, determining water association, proton transfer and hydrogenation process in NO_3_RR. Here, different O−H stretching vibrations from adsorbed water molecules, which are located within the broad band ranging from 2700 to 3800 cm^−1^, were observed for RuO_x_@TAPB‐COF and pristine RuO_2_ (Figure 5f,g, respectively) [11]. Gaussian fitting of the FTIR spectra showed that the O−H stretching band can be deconvoluted into three distinct components, namely: 4‐coordinated hydrogen‐bonded water (4‐HB H_2_O), 2‐coordinated hydrogen‐bonded water (2‐HB H_2_O), and alkali metal‐ionized water (Na H_2_O) [35, 36]. The amount of these interfacial water species at the surface of RuO_2_ showed a positive correlation with the elevated polarizing potential, where the distinct slopes reflected the different dependence of water species on the applied potential (Figure S32). Steeper slope means that 2‐HB H_2_O was more sensitive to the local electric field than 4‐HB H_2_O and Na H_2_O. The proportion that the three water species take was then calculated through integrating the deconvoluted FTIR peaks to analyze how they affect the NO_3_RR kinetics. Though interfacial water molecules may experience dynamic restructuring when applying more negative potential, 4‐HB H_2_O molecules continued to dominate the H‐bond network at RuO_2_ surface (Figure 5h). Prevalence of 4‐HB H_2_O molecules favored the deprotonation of water and the proton transfer from the surface of electrocatalyst to form hydrogen through the Heyrovsky step [37], so that the hydrogenation process of nitrogenous intermediates was inhibited during the NO_3_RR process. Similarly, steeper slope of the curves shown in Figure S33 indicated that in RuO_x_@TAPB‐COF, 2‐HB H_2_O was more sensitive to the local electric field than 4‐HB H_2_O and Na H_2_O. However, 2‐HB H_2_O replacing 4‐HB H_2_O in RuO_x_@TAPB‐COF dominated the interfacial H‐bond network (Figure 5i), implying that the deprotonation of water and proton transfer were partially suppressed. This suggests that the Heyrovsky step was suppressed as well, while it still guaranteed the proton transfer required for the hydrogenation of nitrogenous intermediate in NO_3_RR.

**FIGURE 6:**
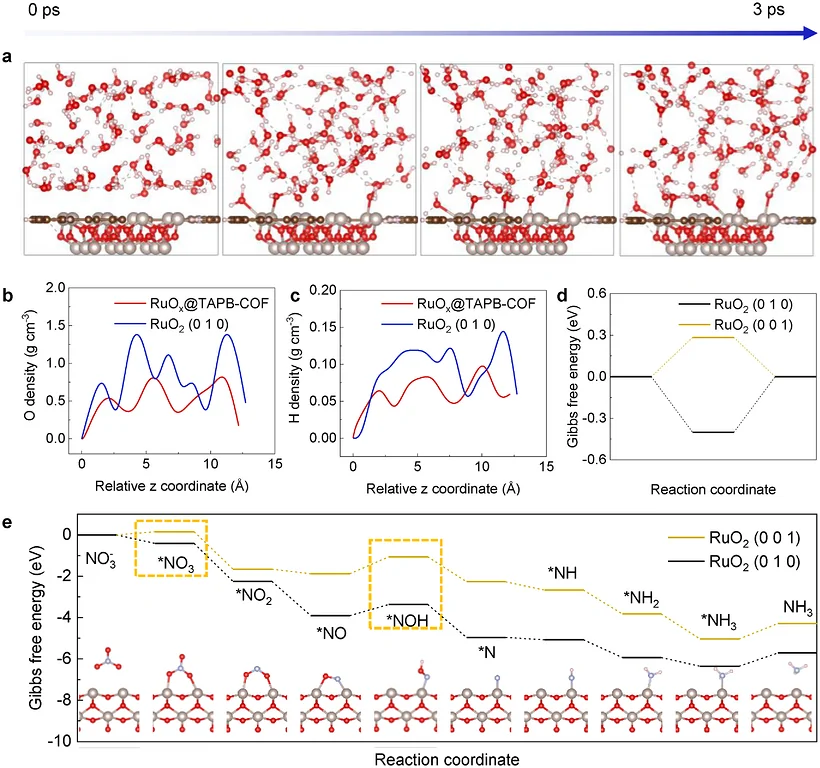
NO_3_RR mechanism analysis based on AIMD and DFT calculations. (a) Snapshots of pre‐adsorbed water molecules at the surface of RuO_x_@TAPB‐COF at 0, 1, 2, and 3 ps, obtained by AIMD. Variation of (b) O and (c) H density upon increasing *z* coordinate starting from the surface of RuO_x_@TAPB‐COF and RuO_2_ (0 1 0) plane. Gibbs free energies of (d) H_2_ formation, and (e) NO_3_RR process over RuO_2_ (0 1 0) and RuO_2_ (0 0 1) planes; adsorption diagrams of nitrogenous intermediates over RuO_2_ (0 1 0) plane are presented too. Color coding for the elements: Ru, brown; O, red; H, white; C, dark brown; N, blue.

Different degree of hydrophilicity of the TAPB‐COF framework and RuO_x_ nanoparticles confined within may contribute to the dominant occupation of 2‐HB H_2_O in the interfacial water network on RuO_x_@TAPB‐COF. Indeed, RuO_2_ nanoparticles were super‐hydrophilic, as evidenced by the small (close to zero) contact angle of water droplets on them (Figure S34a), while the hydrophilicity of TAPB‐COF was much less, as indicated by larger contact angle (31.3°, Figure S34c). Simultaneously, AIMD simulation results (Figure 6a and Figure S36) demonstrated the repelling effect of the hydrophobic TAPB‐COF on water molecules, with a consequence that adsorption of the latter and the hydrogen bond network should be constrained close to the surface of hydrophilic RuO_x_ nanoparticles. According to the oxygen and hydrogen density distribution profiles in Figure 6b,c, the interfacial water was defined as being within 4.0 Å of the surface. Compared with RuO_2_, the distribution of the interfacial oxygen atoms was slightly shifted away from the surface in RuO_x_@TAPB‐COF, while −N═C− and C−O functional groups of TAPB‐COF formed hydrogen bonds with water molecules, thus inducing higher hydrogen density at a low *z* coordinate relative to the surface of RuO_x_ nanoparticles. Thus, the inactivity of TAPB‐COF and the interruption of interfacial water network around RuO_x_ nanoparticles owing to the periodical hydrophobic COF slowed down the rates of proton generation and proton transfer, resulting in a suppressed HER process and an accelerated NO_3_RR process.

Using density functional theory (DFT), we also calculated Gibbs free energies (Figure 6e) of each hydrogenation step in NO_3_RR, as revealed from the spectral analysis (cf. Figure 5a,b and Figure S31a,b). The (0 1 0) and (0 0 1) lattice planes of tetragonal RuO_2_ were separately set as active surfaces for RuO_x_@TAPB‐COF and RuO_2_ in agreement with the above discussed HRTEM (Figure 1d and Figure S10). The CN of Ru on the top layer of RuO_2_ (0 1 0) and RuO_2_ (0 0 1) were 3 and 4, respectively, as illustrated in Figure S37. The ${\mathrm{NO}}_{3}^{-}$ reactant was first chemically absorbed on the Ru site within RuO_2_ (0 1 0) plane to form *NO_3_ adsorbate with a concomitant decrease of total energy, which was indicative of the spontaneity of NO_3_RR reaction. A positive Gibbs free energy (Δ*G*) of 0.15 eV was estimated for Ru site within RuO_2_ (0 0 1) plane, meaning that this should be an endothermic process. Then, the N−O bond experienced cleavage through the proton‐coupled electron transfer to form *NO_2_ and *NO. Thereafter, the *NO intermediate was gradually converted to *NOH, *N, *NH, *NH_2_, and *NH_3_. All these steps were exothermal except the formation of *NOH on both RuO_2_ (0 1 0) and RuO_2_ (0 0 1), which was thus the rate‐determining step of the whole NO_3_RR process. Δ*G* values for the hydrogenation of *NO to *NOH decreased from 0.82 eV on RuO_2_ (0 0 1) to 0.55 eV on RuO_2_ (0 1 0). Notably, stronger binding of *NO_2_ was found in the bridge‐adsorbed *NO_2_ on RuO_2_ (0 1 0) plane containing two shorter Ru−O lengths (O belongs to *NO_2_, 1.99 Å and 2.02 Å), then on RuO_2_ (0 0 1) plane (2.12 Å and 2.12 Å). This was a result of the shorter distance between two adjacent Ru sites (Figure S38), which was 3.11 Å on RuO_2_ (0 1 0) plane, and 4.22 Å on RuO_2_ (0 0 1) plane. This means that the desorption of nitrite by‐product became difficult when bonding at the RuO_2_ (0 1 0) surface. Corresponding d‐band centers of the projected density of states (PDOS) in relation to RuO_2_ (0 1 0) and RuO_2_ (0 0 1) were calculated as ‐0.75 and ‐0.93 eV, respectively. An up‐shift of the center for RuO_2_ (0 1 0) means that the distribution of *d*‐band electrons was moving close to the Fermi level, leading to enhanced adsorption for the critical intermediates *NOH and *NO_2_ (Figure S39). We have also estimated ΔG of HER. As shown in Figure 6d, the exposed Ru site located on the (0 1 0) plane had an absolute ΔG of 0.40 eV, which was much higher than that on RuO_2_ (0 0 1) plane (0.28 eV). Thus, RuO_2_ (0 1 0) plane suppressed the hydrogen generation. Based on all the experimental and theoretical data, we can conclude that the interfacial water network was modulated in the RuO_x_@TAPB‐COF because of the different hydrophilicity between TAPC‐COF and RuO_x_ nanoparticles, leading to an optimal proton transfer rate during NO_3_RR process. Shorter Ru−Ru distance and the upshift of the *d*‐band center in RuO_2_ (0 1 0) contributed to stronger adsorption of *NO_2_ and *NOH intermediates, which induced less nitrite production and accelerated the rate‐determining step of NO_3_RR process.

## 3 Conclusions

We revealed the importance of different hydrophilicity of RuO_x_ nanoparticles and TAPB‐COF on modulating the interfacial hydrogen‐bond water network within RuO_x_@TAPB‐COF composite to achieve the high Faradaic efficiency of ammonia production by electrochemical nitrate reduction reaction. The confined RuO_x_ nanoparticles acted as a “tailor,” sewing up adjacent layers through the coordination with the salicylaldehyde‐imine unit of TAPB‐COF. High oxygen‐deficiency was observed for RuO_x_ nanoparticles due to the confinement effect of COF. RuO_x_@TAPB‐COF showed potential‐dependent Faradaic efficiency reaching maximum value of 99.2% at ‐1.1 V vs Ag/AgCl for ammonia synthesis in neutral electrolyte. In situ FTIR and in situ Raman measurements were conducted to capture the nitrogenous intermediates and determine the interfacial water coordination during NO_3_RR process. Repelling effect of water molecules on hydrophobic TAPB‐COF contributed to the dominant occupation of 2‐H H_2_O at the interfacial water network, which resulted in a modified proton transfer kinetic during NO_3_RR. The upshift of the *d*‐band center and shorter Ru−Ru distance induced strengthened bonding of *NO_2_ and *NOH intermediates which in turn facilitated reduction of *NO in the rate‐determining step (*NO + H^+^ +e → *NOH). This study provided important insights on design of NO_3_RR electrocatalysts with high activity and selectivity based on interfacial microenvironment.

## Conflicts of Interest

The authors declare no conflicts of interest.

## Supporting information




**Supporting File**: advs76147‐sup‐0001‐SuppMat.docx.

## Data Availability

The data that support the findings of this study are available in the Supporting Information of this article.
