# Supplementary material for: Interfacial Water Coordination on Ruthenium Oxide Nanoparticles Confined Within Covalent Organic Framework and Its Effect on Electrochemical Nitrate Reduction
Source: Adv Sci (Weinh). 2026 Jun 17;13(51):e76147. doi: 10.1002/advs.76147 (PMC13336353; doi:10.1002/advs.76147)
Supplement: Supplementary file 1 [file ADVS-13-e76147-s001.docx]

**Supporting Information**

**Interfacial Water Coordination on Ruthenium Oxide Nanoparticles Confined within Covalent Organic Framework and Its Effect on Electrochemical Nitrate Reduction**

*Yun Li,^a^ Xin Zhao,^a,b^ Arsenii S. Portniagin,^a^ Yuxuan Wu,^c^ Desui Chen,^a^ Haochen Liu,^a^ Andrey L. Rogach ^*a^*

^a^ Department of Materials Science and Engineering, City University of Hong Kong, 83 Tat Chee Avenue, Kowloon, Hong Kong S.A.R. 999077, P. R. China

^b^ Department of Materials Science and Engineering, Hainan University, 58 Renmin Avenue, Haikou 570228, P. R. China

^c^ Department of Chemistry, City University of Hong Kong, 83 Tat Chee Avenue, Kowloon, Hong Kong S.A.R. 999077, P. R. China

^*^E-mail: andrey.rogach@cityu.edu.hk

**Experimental Section**

***Characterization***. Powder X-ray diffraction (XRD) patterns were recorded on a Rigaku X-ray diffractometer (SmartLabTM 9kW) with Cu Kα radiation. Scanning electron microscopy (SEM) images were collected on a Thermo Scientific scanning electron microscope (Quattro S) operated at 20 kV. Transmission electron microscopy (TEM) images were obtained on JEM-2100F field emission electron microscope operated at 200 kV. Aberration-corrected transmission electron microscopy (AC-TEM) images, high-angle annular dark field scanning transmission electron microscopy (HAADF-STEM), energy-dispersive X-ray spectroscopy (EDS), and electron energy loss spectroscopy were conducted on a JEOL ARM 300F microscope operating at an accelerating voltage of 300 kV. Fourier transform infrared (FTIR) spectra were recorded on a PerkinElmer Spectrum Two FT-IR spectrometer. The *in-situ* FTIR cell was supplied by Zhongyanhuake Tec. Raman spectra were measured on a WITec Alpha300 Raman Imaging Microscope. Photoluminescence (PL) measurements were carried out on a FLS1000 spectrometer (Edinburgh Instruments). Nitrogen adsorption isotherms were measured at 77 K with a Micromeritics ASAP 2460 analyzer. All samples were degassed under vacuum at 120 ℃ for at least 8 h prior to measurement. The Brunauer-Emmett-Teller (BET) method was used to calculate the specific surface areas using adsorption data in a relative pressure range from 0.025 to 0.150 with R > 0.9999. X-ray photoelectron spectroscopy (XPS) was conducted on a Thermo Fisher ESCALAB250 spectrometer in an ultra-high vacuum using Al Kα X-ray source, which was calibrated by C 1s binding energy (284.8 eV). UV-vis adsorption spectra were collected on a Shimadzu UV-Vis-NIR spectrophotometer (UV-3600). Nuclear magnetic resonance (NMR) spectra were measured on a Bruker Avance NEO operated under 400 MHz. X-ray absorption fine structure spectra for the Ru K-edge were measured on the BL14W1 line station of the Shanghai Synchrotron Radiation Facility in a transmission mode.

***Chemicals***. All chemicals were of analytical grade and used without further purification. 1,3,5-tris(4-aminophenyl)benzene (TAPB), 2-hydroxy-1,3,5-triformylbenzene (HTFB), 1,2-diaminocyclohexane, 1,2-dichlorobenzene, sodium hydroxide, sodium citrate, sulfanilamide, N-(1-Naphthyl) ethylenediamine dihydrochloride, sodium hypochlorite, sodium nitroprusside, and sodium borohydride were purchased from Aladdin Scientific Corp. Glacial acetic acid, phosphoric acid, salicylic acid, n-butanol, poly-N-vinyl-2-pyrrolidone and methanol were purchased from Sigma-Aldrich. Ruthenium chloride was purchased from Macklin Inc., while reference RuO_2_ electrocatalysts were purchased from Sinero Tech. Corp.

***Synthesis of TAPB-COF***. 35.8 mg of HTFB and 22.5 mg of 1,2-diaminocyclohexane were added into a three-necked flask filled with the absolute alcohol under continuous stirring. The flask was subjected to degasification procedure on a Schlenk line 3 times, and then gradually heated up and kept at 70 ℃ for 12 h. After cooling down to room temperature, the yellow product was collected by centrifugation at 5000 rpm. After washing with absolute alcohol 3 times, the final product was dried in a vacuum oven at 40 ℃ for 24 h and dispersed in a mixture of 6.4 mL 1,2-dichlorobenzene and 1.6 mL n-butanol.

To synthesize TAPB-COF, 2 mL of the precursor synthesized above and 5.8 mg of TAPB were added into a flask and diluted using 6 mL of the above prepared mixture. To trigger the Schiff base reaction, 600 μL of glacial acetic acid and 300 μL of ultra-pure water were added into the same flask, which was heated to 120 ℃ and kept at this temperature for 3 days. The brick-red product was washed with acetone and methanol for several times, collected by centrifugation and dried in a vacuum oven at 60 ℃ for 24 h.

***Synthesis of RuO_x_@TAPB-COF***. To synthesize RuO_x_@TAPB-COF, 5 mg TAPB-COF was first dispersed in 700 mL mixture of methanol and water, and 10.89 mg of RuCl_3_$\cdot$xH_2_O was added. The mixture was heated to 70 ℃, and kept at this temperature for 12 h. The dark-red product was washed with deionized water and methanol for several times, and collected by centrifugation. We also prepared a series of RuO_x_@TAPB-COF using the same method except for taking varying amounts of RuCl_3_$\cdot$xH_2_O precursor: 5.44, 2.72, 1.36, and 0.68 mg, respectively. The measured actual Ru amount (wt.%) for these samples was 7.46%, 2.80%, 2.18%, 1.51%, and 1.28%, respectively, as determined by inductively coupled plasma spectrometry.

***Electrochemical Measurements***. Electrochemical performance of the nitrate reduction reaction (NO_3_RR) was evaluated in a H-type electrolytic cell. 0.1 M Na_2_SO_4_ solution was used as electrolyte in the anode compartment, while the mixture solution of 0.1 M Na_2_SO_4_ and 0.1 M NaNO_3_ was used in the cathode counterpart. Pt plate and Ag/AgCl electrode were used as a counter electrode and a reference electrode, respectively. 2.5 mg of the electrocatalyst and 2.5 mg of carbon black were added in a mixture of 100 μL of Nafion 117 solution and 900 μL of methanol. After ultrasonication, 600 μL of the prepared ink was dropped onto the carbon felt (working area: 0.5$\text{×}$1 cm^2^). Linear sweep voltammetry (LSV) curves were measured at a scanning rate of 5 mV s^-1^ in the potential range of -0.2 ~ -1.5 V vs. Ag/AgCl. Cyclic voltammetry (CV) curves were measured at a potential range of -0.5 ~ -0.4 V vs. Ag/AgCl with varying scanning rate to calculate electrochemical capacitance. Potentiostatic tests were conducted at different potentials for 0.5 h with a stirring rate of 350 rpm.

***Assembly of Zinc-Nitrate Batteries***. 2.5 mg of the RuO_x_@TAPB-COF and 2.5 mg of carbon black were added into a mixture of 100 μL of Nafion 117 solution and 900 μL of methanol, and the ink was ultrasonicated for 30 min. For comparison, 2.5 mg of the commercial RuO_2_ electrocatalyst and 2.5 mg of carbon black were used to prepare the reference electrodes in a similar manner. To fabricate the cathode, 600 μL of the ink was dropped onto the carbon felt (working area: 0.5$\text{×}$1 cm^2^) and dried. Before the assembly of the zinc-nitrate battery, the zinc plate was polished with a sandpaper to remove any traces of the oxide layer from the surface. The zinc-nitrate battery was assembled using 0.25 M NaNO_3_ and 5 M LiCl as a cathode electrolyte and 5 M KOH as an anode electrolyte.

***Determination of Concentration***. A UV-Vis spectrophotometry was used to determine the concentration of nitrite and ammonium. The products were taken out from the cathode compartment and diluted to detection range.

*Determination of Nitrite*. The chromogenic reagent for nitrate determination was obtained by mixing sulfanilamide (4 g), N-(1-Naphthyl) ethylenediamine dihydrochloride (0.2 g), ultrapure water (50 mL) and phosphoric acid (10 mL). Then, 0.1 mL of the chromogenic reagent was added to 5 mL of diluted electrolyte. The absorbance spectrum was recorded in the wavelength range of 500 ~ 600 nm, and the absorption intensity at 540 nm was recorded after resting for 20 min. The calibration was performed using the Griess reagent method with a series of NaNO_2_ solutions of a known concentration.

*Determination of Ammonia*. The chromogenic reagent for detecting ammonia contained the following three parts (all aqueous solutions). The first part contained 5 wt.% salicylic acid, 5 wt.% sodium citrate, and 1 M NaOH; the second one was the mixture of 0.05 M NaClO and 2 M NaOH; and the third one was 1 wt.% sodium nitroprusside solution. 2 mL, 1 mL and 0.2 mL of the above three parts of the chromogenic reagent were subsequently added to 2 mL of diluted electrolyte. The absorbance spectrum was recorded in the wavelength range of 500 ~ 800 nm. After allowing the mixture to stand for 2 h to ensure the complete color development, the absorption intensity at 655 nm was recorded for quantitative analysis. The calibration was performed using the indophenol blue method with a series of (NH_4_)_2_SO_4_ solutions of a known concentration.

***^15^N Isotope Labeling Experiments*.** To clarify the origin of ammonia, isotope labeled nitrate reduction experiments were conducted by using Na^14^NO_3_ and Na^15^NO_3_ as the nitrogen source. After chronoamperometry tests at -1.5 V vs. Ag/AgCl for 0.5 h, the electrolyte from cathodic compartment was collected to perform NMR measurements.

***Collection of NH_4_NO_3_***. We used a simple apparatus to collect the ammonia gas generated during the NO_3_RR process. The argon flow was introduced into the electrolyzer system from one side of the cathode compartment, so that the generated ammonia could be carried into another conical flask containing 0.1 M nitric acid solution. The obtained solution was concentrated in a rotary evaporator at 50 ℃. White powder of NH_4_NO_3_ was obtained by evaporating the solvent in an oven at 60 ℃.

***In-situ* *FTIR Measurements*.** FTIR device consisted of an IR spectrometer and an IR microscope (Bruker INVENIO) with a 16× objective, which enabled measurements over a broad range of 15 ~ 4000 cm^−1^ with a high spectral resolution (0.25 cm^−1^). In the *in-situ* FTIR cell, silica was used as the transmission window. The measured potentials for NO_3_RR were set in a range from -0.2 V to -1.4 V vs. Ag/AgCl, and the spectra were acquired after applying potential for 5 min. The background spectrum of the electrocatalyst electrode was acquired at an open-circuit voltage before each measurement.

***In-situ Raman Spectroscopy***. *In-situ* Raman spectra were measured in an electrochemical cell (Shanghai Chuxi) in a horizontal configuration allowing for irradiation from the top, using excitation wavelength of a He-Ne laser (532 nm). Spectra were recorded in the range of 845 ~ 1900 cm^-1^ with an acquisition time of 10 s and accumulation of 10 spectra.

***NMR Spectroscopy*.** The pH of the electrolyte collected after reaction was adjusted to 2 by adding 0.1 M HCl. 0.5 mL of the sample solution was mixed with 0.1 mL of DMSO-d_6_ (hexadeuterodimethyl sulfoxide) containing 0.04 wt.% maleic acid as the internal standard.

***Estimation of the Fermi Level***. The Shirley background was first subtracted from the measured valence band spectrum. The Fermi level was estimated by subtracting the He I radiation energy (21.2 eV) from the high-binding energy cut-off of the ultraviolet photoelectron spectra.

***AIMD Simulations.*** To obtain the precise atomic structure of the interfacial water, we employed *ab initio* molecular dynamics (AIMD) method to simulate the interfaces of RuO_x_@TAPB and RuO_2_ interacting with water molecules. The simulation was performed by means of density functional theory (DFT) using the CP2K pack. CP2K/Quickstep used two representations of the electron density: localized Gaussian and plane wave basis sets. For the Gaussian-based (localized) expansion of the Kohn–Sham orbitals, we used a library of the contracted molecularly optimized valence double-zeta plus polarization basis sets;^1^ the complementary plane wave basis set had a cut-off of 400 Rydberg for the electron density. The generalized gradient corrected approximation of the Perdew-Burke-Ernzerhof (PBE) method was adopted to relax the geometric structures.^2^ The dispersion correction was applied in all calculations with the Grimme D3 method.^3^ For the sampling of the structures of bulk solution, the canonical ensemble (NVT) conditions were imposed by a Nose-Hoover thermostat with a temperature of 300 K.

***DFT Calculations***. DFT calculations were performed via the Vienna *ab-initio* Simulation Package (VASP) using the PBE method. We used the plane wave basis with a cut-off energy of 450 eV, electronic self-consistent field 1$\times$10^-5^ eV, ionic convergence energy 0.02 eV Å^-1^, and Gaussian electron smearing width 0.05 eV for geometry optimization. We calculated the Gibbs free energies $\Delta G$ for different nitrogenous intermediates involved in the NO_3_RR process:

$$* + {NO}_{3}^{-} \to*{NO}_{3} + e \Delta G1$$

$$*{NO}_{3} + 2H^{+} + 2e \to*{NO}_{2} +H_{2}O \Delta G2$$

$$*{NO}_{2} + 2H^{+}+ 2e \to*NO + H_{2}O \Delta G3$$

$$*NO + H^{+} + e \to*NOH \Delta G4$$

$$*NOH + H^{+} + e \to*N + H_{2}O \Delta G5$$

$$*N + H^{+}+ e \to*NH \Delta G6$$

$$*NH + H^{+}+ e \to*{NH}_{2} \Delta G7$$

$$*{NH}_{2} + H^{+}+ e \to*{NH}_{3} \Delta G8$$

The overall reaction:

$$\mathrm{NO}_{3}^{-}+8e+9H^{+}\to{NH}_{3}+3H_{2}O$$

The Gibbs free energy of each elementary step was calculated as:

$$\Delta G= \Delta E+ \Delta ZPE-T\Delta S$$

where $\Delta E$ represents the reaction energy, while $\Delta ZPE$ and $T\Delta S$ represent two correction terms – zero-point energy and entropy. $\Delta E$ was calculated by subduction of total electronic energy of products and reactants, and $\Delta ZPE$ and $T\Delta S$ were derived from the vibration frequency of the phonons.

**Figure S1**. Synthesis of the RuO_x_@TAPB-COF composite.

**Figure S2**. Simulated structures for TAPB-COF and RuO_x_@TAPB-COF; TAPB-COF has a triclinic lattice structure with parameters of a = 17.5 Å, b = 17.5 Å, c = 3.7 Å, α = 90 °, β = 90 °, and γ = 120 °. The calculated lattice spacing of (1 0 0) and (2 1 0) for TAPB-COF are 15.2 Å and 8.8 Å after geometric optimization, respectively. Color coding for the elements: Ru, brown; O, red; H, white; C, dark brown; N, light blue.

**Figure S3**. SEM image of TAPB-COF.

**Figure S4**. HAADF-STEM image of TAPB-COF.

**Figure S5**. FTIR spectra of HTFB, TAPB, TAPB-COF and RuO_x_@TAPB-COF.

**Figure S6**. Size distribution of RuO_x_ nanoparticles confined within TAPB-COF. The black curve is a Gaussian fit.

**Figure S7**. TEM image of RuO_x_@TAPB-COF.

**Figure S8**. HRTEM image of RuO_x_@TAPB-COF.

**Figure S9**. HAADF-STEM image of RuO_x_@TAPB-COF. The observed lattice spacing of 0.220 nm in RuO_x_ nanoparticles belongs to the (2 0 0) plane of RuO_2_ tetragonal phase.

**Figure S10**. (a) TEM and (b) HRTEM image taken from the yellow-square marked region in (a) of RuO_2_ nanoparticles with larger size. The interplanar distance of (1 1 0) is shown in (b).

**Figure S11**. (a) Atomic resolution HADDF-STEM image of RuO_x_@TAPB-COF. (b) Electron energy loss spectra taken from two different regions (1 and 2) of RuO_x_@TAPB-COF. Yellow squares (1) and (2) represent the areas with and without nanoparticles in RuO_x_@TAPB-COF, revealed by the respective spectra.

**Figure S12**. Raman spectra of RuO_2_, TAPB-COF, and RuO_x_@TAPB-COF. The characteristic vibration observed around 560 cm^-1^ is assigned to Ru−O(N), showing the coordination of Ru within RuO_x_@TAPB-COF.

**Figure S13**. SEM images of RuO_x_@TAPB-COF samples with different Ru amount (wt. %) of (a) 7.46%, (b) 2.80%, (c) 2.18%, (d) 1.51%, and (e) 1.28%, respectively.

**Figure S14**. The lattice spacing of (0 0 1) planes of RuO_x_@TAPB-COF samples as a function of Ru loading (wt. %), determined by inductively coupled plasma spectrometry.

**Figure S15**. PL spectra of RuO_x_@TAPB-COF samples with different Ru amount (wt.%) of 1.28%, 1.51%, 2.18%, 2.80%, and 7.46%. All the samples are measured using the same concentration of 0.18 mg mL^-1^; the excitation wavelength is 400 nm.

**Figure S16**. (a) Nitrogen adsorption-desorption isotherms, and (b) pore size distribution curves of TAPB-COF and RuO_x_@TAPB-COF with Ru amount of 7.46 wt.%.

**Figure S17**. High-resolution XPS spectra of C 1s and Ru 3d orbitals of RuO_2_, RuO_x_@TAPB-COF, and TAPB-COF. The Ru 3d XPS spectra were deconvoluted into three pair of peaks assigned to Ru^3+^, Ru^4+^, and the satellite peak in cases of pristine RuO_2_ and RuO_x_@TAPB-COF, where a negative shift occurs in RuO_x_@TAPB-COF compared with RuO_2_ as indicated by the vertical lines.

**Figure S18**. High-resolution XPS spectra of N 1s orbitals of RuO_x_@TAPB-COF and TAPB-COF.

**Table S1.** Ru K-edge EXAFS analysis for RuO_2_ and RuO_x_@TAPB-COF.

|  | Path | CN | R (Å) | σ^2^ (10^-3^Å^2^) | ∆E_0_ (eV) | R-factor |
| --- | --- | --- | --- | --- | --- | --- |
| RuO_2_ | Ru−O | 5.3±0.2 | 1.96±0.02 | 3.0±0.4 | -2.5±1.2 | 0.0180 |
| RuO_x_@TAPB-COF | Ru−O | 3.2±0.2 | 1.99±0.02 | 4.5±0.5 | 2.2±1.1 | 0.0154 |

CN, coordination number; R, distance between the absorber and backscatter atoms; σ^2^, Debye–Waller factor to account for both thermal and structural disorders; ΔE_0_ (eV), inner potential correction to account for the difference in the inner potential between the sample and the reference compound; R-factor indicates the goodness of the fit. The amplitude reduction factor (S_0_^2^) was set to 1.0 during the fitting process.

**Figure S19**. Linear sweep curves of RuO_x_@TAPB-COF measured in 0.1 M Na_2_SO_4_ electrolyte with and without 0.1 M NaNO_3_.

**Figure S20**. (a) UV-vis absorption spectra for different concentrations of ammonium chloride obtained by the indophenol blue method, and (b) calibration curves of ammonia based on those UV-vis absorption spectra. The inset in (b) shows the color-changing trend of (NH_4_)_2_SO_4_ solutions with elevating concentration after adding chromogenic reagent.

**Figure S21**. (a) UV-vis absorption spectra for different concentrations of sodium nitrite obtained by the Griess reagent method, and (b) calibration curves for nitrite based on those UV-vis absorption spectra. The inset in (b) shows the color-changing trend of NaNO_2_ solutions with elevating concentration (from 0 mg/L to 1.2 mg/L) after adding chromogenic reagent.

**Figure S22**. Comparison of the NO_3_RR performance demonstrated in this work (red star) with other reported electrocatalysts measured under neutral conditions.^4-12^

**Figure S23**. CV curves of (a) RuO_2_, (b) RuO_x_@TAPB-COF, and (c) TAPB-COF measured at the potential range from 2 mV s^-1^ to 12 mV s^-1^. (d) Plots of current densities (at -0.45 V vs. Ag/AgCl) as a function of scan rates for the above three samples. Electrochemical capacitances were calculated to be 176.4 mF cm^-2^, 165.8 mF cm^-2^, and 209.2 mF cm^-2^ for RuO_2_, RuO_x_@TAPB-COF, and TAPB-COF, respectively.

**Figure S24**. Normalized yield rate of ammonium for RuO_2_ and RuO_x_@TAPB-COF which considered the electrochemically active surface area (ECSA) measured in 0.1 M Na_2_SO_4_ with addition of 0.1 M NaNO_3_.

The yield rates of RuO_2_ at -1.3 V and -1.4 V vs. Ag/AgCl were slightly higher than that of RuO_x_@TAPB-COF. This is because of the large ECSA of RuO_x_@TAPB-COF, which originated from TAPB-COF as verified in **Fig. S23d**. However, the observed low Faradaic efficiency of ammonia of pristine TAPB-COF (**Fig. S25**), which was less than 16% in the potential ranging from -1.0 V to -1.5 V vs. Ag/AgCl, reflects the poor NO_3_RR activity in this case.

**Figure S25**. Faradaic efficiency and yield rate of ammonia (red) and nitrite (blue) determined for pristine TAPB-COF electrocatalyst.

The Faradaic efficiency for ammonia and nitrite decreased when the applied potential increased from -1.0 V to -1.5 V vs. Ag/AgCl, while the yield rate of nitrite increased from 0.06 mmol mg^-1^ h^-1^ to 0.08 mmol mg^-1^ h^-1^. This reveals the poor NO_3_RR activity for pristine TAPB-COF.

**Figure S26**. Faradaic efficiency and yield rate of ammonia as a function of the Ru amount (wt. %) for RuO_x_@TAPB-COF samples at -1.1 V vs. Ag/AgCl.

The curve showed a positive correlation between the Ru amount and the yield of ammonia under a constant potential. The Faradaic efficiency increased from 18.0% to 99.2% as the Ru content increased from 1.28 wt.% to 7.46 wt.%, while the yield rate increased from 0.01 to 0.11 $\mathrm{mmol}\mathrm{mg}_{\mathrm{Ru}}^{-1} h^{-1}$.

**Figure S27**. ^1^H NMR spectra of RuO_x_@TAPB-COF after reaction at -1.5 V vs. Ag/AgCl while feeding Na^14^NO_3_ and Na^15^NO_3_ as nitrogen sources.

**Figure S28**. Duration curves of RuO_x_@TAPB-COF measured under a constant potential of -1.1 V vs. Ag/AgCl.

**Figure S29**. Static open circuit potential curves of zinc-nitrate batteries based on RuO_2_ or RuO_x_@TAPB-COF cathodes.

**Figure S30**. Specific capacities of zinc-nitrate batteries based on RuO_2_ or RuO_x_@TAPB-COF cathodes, measured at 0.5 mA cm^-2^.

**Figure S31**. *In-situ* Raman spectra of (a) RuO_x_@TAPB-COF and (b) RuO_2_ measured under open circuit potential (OCP), and at polarizing potentials in a range from -0.5 to -0.9 V vs. Ag/AgCl.

**Figure S32**. Variation of the peak area of interfacial water (4-HB H_2_O, 2-HB H_2_O, and Na H_2_O) on RuO_2_ versus applied potential.

**Figure S33**. Variation of the peak area of interfacial water (4-HB H_2_O, 2-HB H_2_O, and Na H_2_O) on RuO_x_@TAPB-COF versus applied potential.

**Figure S34**. Shape of water droplets on (a) RuO_2_, (b) RuO_x_@TAPB-COF, and (c) TAPB-COF, with the contact angles of ~ 0 °, 26.5 °, and 31.3 °, respectively.

**Figure S35**. Structure model of RuO_x_@TAPB-COF used for AIMD. Color coding for the elements: Ru, brown; O, red; H, white; C, dark brown; N, light blue.

**Figure S36**. Snapshots of pre-adsorbed water molecules on the RuO_2_ (0 1 0) surface at 0, 1, 2, 3, 4 and 5 ps, simulated using AIMD. Color coding for the elements: Ru, brown; O, red; H, white.

**Figure S37**. Structure models of RuO_2_ (0 1 0) and RuO_2_ (0 0 1) slabs. CN stays for coordination number. Color coding for the elements: Ru, brown; O, red; H, white.

**Figure S38**. Structure models of *NO_2_ intermediate absorbed on RuO_2_ (0 0 1) and RuO_2_ (0 1 0). Ru−Ru and Ru−O (O belongs to *NO_2_) distances are indicated. Color coding for the elements: Ru, brown; O, red; H, white.

**Figure S39**. Projected density of state (PDOS) of the *d*-bands of RuO_2_ (0 1 0) and RuO_2_ (0 0 1); the corresponding *d*-band centers are indicated by the dashed lines, RuO_2_ (0 1 0) has a higher value of -0.75 eV than that of RuO_2_ (0 0 1) (-0.93 eV).

**References**

1.	VandeVondele, J.; Hutter, J., Gaussian basis sets for accurate calculations on molecular systems in gas and condensed phases. *J Chem Phys* **2007,** *127* (11), 114105.

2.	Blochl, P. E., Projector augmented-wave method. *Phys Rev B Condens Matter* **1994,** *50* (24), 17953-17979.

3.	Grimme, S.; Ehrlich, S.; Goerigk, L., Effect of the damping function in dispersion corrected density functional theory. *J Comput Chem* **2011,** *32* (7), 1456-65.

4.	Zhang, R.; Zhang, Y.; Xiao, B.; Zhang, S.; Wang, Y.; Cui, H.; Li, C.; Hou, Y.; Guo, Y.; Yang, T.; Fan, J.; Zhi, C., Phase Engineering of High-Entropy Alloy for Enhanced Electrocatalytic Nitrate Reduction to Ammonia. *Angew Chem Int Ed Engl* **2024,** *63* (35), e202407589.

5.	Wei, Y.; Huang, J.; Chen, H.; Zheng, S. J.; Huang, R. W.; Dong, X. Y.; Li, L. K.; Cao, A.; Cai, J.; Zang, S. Q., Electrocatalytic Nitrate Reduction on Metallic CoNi-Terminated Catalyst with Industrial-Level Current Density in Neutral Medium. *Adv Mater* **2024,** *36* (30), e2404774.

6.	Xiong, Y.; Sun, M.; Wang, S.; Wang, Y.; Zhou, J.; Hao, F.; Liu, F.; Yan, Y.; Meng, X.; Guo, L.; Liu, Y.; Chu, S.; Zhang, Q.; Huang, B.; Fan, Z., Atomic Scale Cooperativity of Alloy Nanostructures for Efficient Nitrate Electroreduction to Ammonia in Neutral Media. *Advanced Functional Materials* **2024,** *35* (14).

7.	Sun, L.; Liu, B., Mesoporous PdN Alloy Nanocubes for Efficient Electrochemical Nitrate Reduction to Ammonia. *Adv Mater* **2023,** *35* (1), e2207305.

8.	Wu, Z. Y.; Karamad, M.; Yong, X.; Huang, Q.; Cullen, D. A.; Zhu, P.; Xia, C.; Xiao, Q.; Shakouri, M.; Chen, F. Y.; Kim, J. Y. T.; Xia, Y.; Heck, K.; Hu, Y.; Wong, M. S.; Li, Q.; Gates, I.; Siahrostami, S.; Wang, H., Electrochemical ammonia synthesis via nitrate reduction on Fe single atom catalyst. *Nat Commun* **2021,** *12* (1), 2870.

9.	Liu, H.; Lang, X.; Zhu, C.; Timoshenko, J.; Ruscher, M.; Bai, L.; Guijarro, N.; Yin, H.; Peng, Y.; Li, J.; Liu, Z.; Wang, W.; Cuenya, B. R.; Luo, J., Efficient Electrochemical Nitrate Reduction to Ammonia with Copper-Supported Rhodium Cluster and Single-Atom Catalysts. *Angew Chem Int Ed Engl* **2022,** *61* (23), e202202556.

10.	Zhao, R.; Yan, Q.; Yu, L.; Yan, T.; Zhu, X.; Zhao, Z.; Liu, L.; Xi, J., A Bi-Co Corridor Construction Effectively Improving the Selectivity of Electrocatalytic Nitrate Reduction toward Ammonia by Nearly 100. *Adv Mater* **2023,** *35* (48), e2306633.

11.	Wang, Y.; Hao, F.; Xu, H.; Sun, M.; Wang, X.; Xiong, Y.; Zhou, J.; Liu, F.; Hu, Y.; Ma, Y.; Meng, X.; Guo, L.; Wang, C.; Shao, M.; Wang, G.; Wang, J.; Lu, P.; Yin, J.; Wang, J.; Niu, W.; Ye, C.; Zhang, Q.; Xi, S.; Huang, B.; Shao, M.; Fan, Z., Interfacial Water Structure Modulation on Unconventional Phase Non-Precious Metal Alloy Nanostructures for Efficient Nitrate Electroreduction to Ammonia in Neutral Media. *Angew Chem Int Ed Engl* **2025,** *64* (28), e202508617.

12.	Zhang, M.; Cheng, X.; Duan, Y.; Chen, J.; Wang, L.; Wang, Y. Q., Hydration‐effect Boosted Active Hydrogen Facilitates Neutral Ammonia Electrosynthesis from Nitrate Reduction. *Advanced Functional Materials* **2024,** *35* (2).
